# Primary vaginal adenocarcinoma as a radiation-associated secondary malignancy: A case report and comprehensive literature review

**DOI:** 10.1097/MD.0000000000044272

**Published:** 2025-10-03

**Authors:** Guangyang Xing, Yani Lu, Cui Zhang, Xiaotong Sun

**Affiliations:** a Department of Obstetrics and Gynecology, Gansu Provincial Hospital, Lanzhou, Gansu, China; b The First Clinical Medical College of Gansu University of Chinese Medicine, Lanzhou, Gansu, China; c Department of Pathology, Gansu University of Chinese Medicine – Affiliated Hospital, Lanzhou, Gansu, China.

**Keywords:** case report, immunohistochemical phenotype, non-DES-associated primary vaginal adenocarcinoma, post-chemoradiation carcinogenesis, radiation-induced secondary malignancy

## Abstract

**Rationale:**

Primary vaginal adenocarcinoma (PVA) is an exceptionally rare malignancy, with most cases historically linked to in utero diethylstilbestrol (DES) exposure. Non–DES-associated PVA following definitive chemoradiotherapy for cervical cancer is extremely uncommon, and no standardized management protocol exists. This case illustrates a rare instance of radiation-associated PVA and highlights therapeutic considerations in previously irradiated pelvic fields.

**Patient concerns:**

A 53-year-old postmenopausal woman presented with intermittent vaginal bleeding occurring 5 years after radical hysterectomy and adjuvant chemoradiotherapy for stage IIA2 cervical squamous cell carcinoma.

**Diagnoses:**

Colposcopy-guided biopsies of the vaginal anterior wall revealed glandular architecture with cytological atypia. Immunohistochemistry demonstrated CK7(+), CK8/18(+), CEA(+), PAX8(−), ER(−), PR(−), p16 patchy weak negative, and wild-type p53. Systemic workup excluded metastatic disease, supporting the diagnosis of PVA (non–DES-associated, radiation-related).

**Interventions:**

In view of prior pelvic irradiation, the multidisciplinary team recommended dose-adjusted paclitaxel–cisplatin combination chemotherapy. Six cycles were administered, tailored to minimize cumulative toxicity.

**Outcomes:**

At 6-month follow-up, imaging studies confirmed complete clinical remission, with no locoregional recurrence or distant metastases.

**Lessons:**

This report underscores the potential for radiation-induced secondary vaginal adenocarcinoma in cervical cancer survivors, even after prolonged remission. Vigilant long-term surveillance, including annual vaginal cytology and targeted biopsies, is critical for early detection. Individualized therapeutic strategies – integrating prior treatment history, immunohistochemical profiling, and consideration of molecular alterations – are essential for optimizing outcomes in this rare patient population.

## 1. Introduction

Primary vaginal adenocarcinoma (PVA) is a rare malignant tumor accounting for 1% to 2% of female genital tract malignancies and <10% of all primary vaginal carcinomas.^[1]^ Up to 70% of non-clear cell PVA cases correlate with prenatal diethylstilbestrol (DES) exposure.^[2]^ Nevertheless, contemporary data reveal an increasing incidence of non-DES-associated PVA, particularly among younger women, potentially linked to persistent high-risk human papillomavirus infection.^[3]^ For PVA management, the International Federation of Gynaecology and Obstetrics recommends radical surgery for stage I to II disease, whereas advanced stages (III–IV) warrant external beam radiotherapy combined with intracavitary brachytherapy, with total radiation doses of 70 to 84 Gy required to overcome inherent radioresistance.^[4–6]^

No standardized protocol exists for managing radiation-associated PVA following cervical squamous cell carcinoma treatment. Literature review indicates a 0.5% to 2% cumulative risk of secondary vaginal carcinoma after cervical cancer radiotherapy.^[7]^ Radiation-induced DNA double-strand breaks and *TP53* mutations may drive secondary carcinogenesis, though direct evidence linking radiotherapy dosage to adenocarcinoma development remains elusive.^[8]^

This study presents the first documented case of non-DES-associated PVA occurring 5 years post radical hysterectomy with adjuvant concurrent chemoradiation for cervical cancer. The patient achieved complete remission through paclitaxel-containing combination chemotherapy. This case addresses the evidence gap in PVA management among cervical cancer survivors and implicates radiation-induced tumor microenvironment alterations (hypoxia, fibroblast activation) as potential drivers of secondary malignancies such as vaginal adenocarcinoma.

## 2. Case report

**Figure 1.**
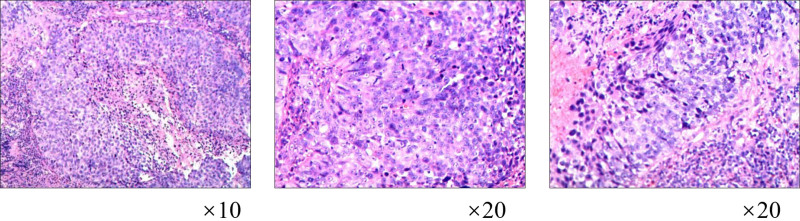
Pathological examination of the uterine specimen after hysterectomy revealed a highly suggestive infiltrating non-keratinizing squamous cell carcinoma of the cervix, stage IIA2 according to FIGO classification. FIGO = International Federation of Gynaecology and Obstetrics.

A 53-year-old postmenopausal woman presented with intermittent abnormal vaginal bleeding 4 years after menopause without family history of malignancy. Five years prior, she had been diagnosed with cervical squamous cell carcinoma (International Federation of Gynaecology and Obstetrics stage IIA2). Her initial treatment consisted of 1 cycle of cisplatin–paclitaxel neoadjuvant chemotherapy followed by radical hysterectomy with bilateral salpingectomy and systematic pelvic lymphadenectomy. Histopathological examination revealed invasive large cell, non-keratinizing squamous cell carcinoma (Fig. 1) with extensive cervical stromal invasion (17 mm/18 mm), lymphovascular space invasion, and involvement of the internal cervical os and bilateral parametria. The vaginal margin, bilateral fallopian tubes, ovaries, and all pelvic lymph nodes were negative for malignancy. Following National Comprehensive Cancer Network guidelines, the patient received adjuvant concurrent chemoradiotherapy comprising image-guided external beam radiation therapy (6MV-X rays) to the pelvic lymphatic drainage area, tumor bed, and proximal vagina (PTV1: 50 Gy in 25 fractions) and bilateral parametria (PTV2: 60 Gy in 30 fractions), high-dose-rate intracavitary brachytherapy (iridium-192, 15 Gy in 3 fractions), and 6 cycles of cisplatin–paclitaxel chemotherapy. Regular follow-up evaluations at 6-month intervals, including pelvic magnetic resonance imaging, computed tomography, and tumor marker assessments, demonstrated sustained complete clinical remission throughout the 5-year posttreatment period.

**Figure 2.**
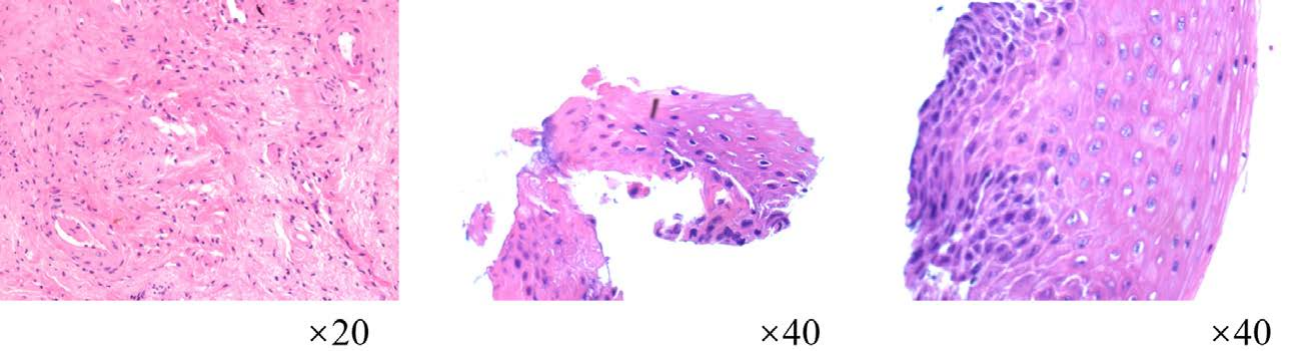
Pathologic examination of the vaginal stump specimen (postoperative for cervical carcinoma) reveals focal residual squamous epithelial cells with no evidence of dysplasia or malignancy.

**Figure 3.**
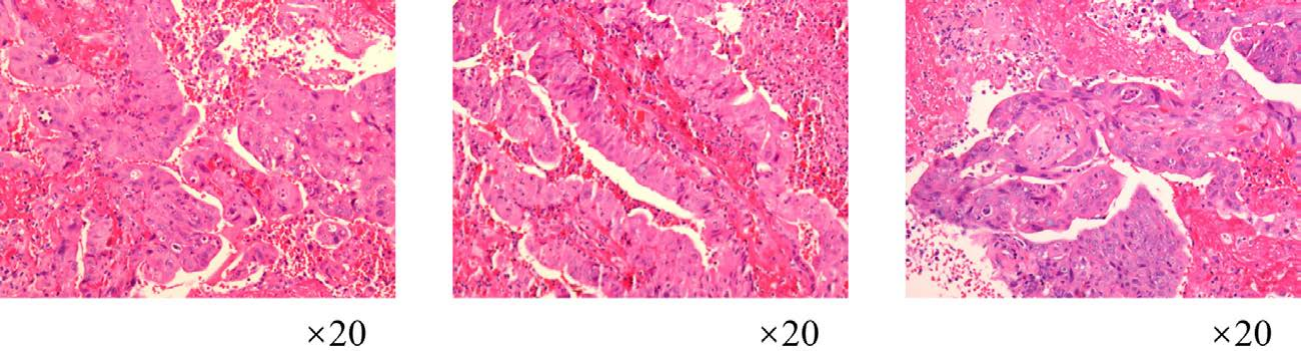
The vaginal wall tissue biopsy showed vaginal adenocarcinoma. Immunohistochemical staining results revealed: CK8/18 (+); CK7 (+); vimentin (−); P16 (−); PAX8 (−); P53 (wild-type pattern); ER (−); PR (−); CEA (−); Ki-67 (+60%).

At 5 years postoperatively, the patient developed 2 episodes of spontaneous vaginal bleeding. Multisite colposcopy-guided biopsies showed no squamous cell carcinoma recurrence at the vaginal stump (Fig. 2). However, vaginal anterior wall lesions demonstrated glandular architecture with cytological atypia. Immunohistochemistry revealed: CK7(+), CEA (+), Pax-8(−), ER (−), PR (−), and patchy weak p16 negative (Fig. 3) (a profile discordant with cervical adenocarcinoma’s typical diffuse strong p16 expression. Systemic evaluation) including contrast-enhanced abdominal MRI, bilateral breast Doppler ultrasonography, chest CT, and gastrointestinal endoscopy (identified no primary lesions, confirming PVA [non-DES-associated subtype]).

Following multidisciplinary team (MDT) consensus (accounting for prior pelvic irradiation) dose-adjusted paclitaxel–cisplatin combination chemotherapy was initiated. Six-month follow-up revealed no evidence of local recurrence or distant metastases.

The patient’s informed consent and exceptional adherence to the MDT-guided therapeutic regimen represent a pivotal strength of this case report. Following rigorous MDT consensus, the decision to administer modified-dose paclitaxel–cisplatin combination chemotherapy (tailored to mitigate potential cumulative toxicity from prior pelvic irradiation) reflects a judicious, patient-centric approach that aligns with current oncological principles for managing radiation-exposed populations. The patient’s unwavering compliance with both the chemotherapeutic protocol and posttreatment surveillance underscores the critical role of patient engagement in optimizing therapeutic outcomes.

The absence of locoregional recurrence or distant metastasis at the 6-month follow-up, validated by contrast-enhanced cross-sectional imaging, is clinically significant. This outcome not only corroborates the efficacy of the adapted regimen but also emphasizes the importance of individualized dose modulation in patients with prior radiotherapy.

## 3. Discussion

PVA remains exceptionally rare, with >90% of reported cases historically linked to in utero DES exposure.^[9]^ However, non-DES-associated PVA incidence demonstrates a rising trend in recent epidemiological reports (particularly among younger populations) a phenomenon potentially attributable to persistent chronic inflammatory stimulation and underlying genomic instability.^[10]^ Current International Federation of Gynecology and Obstetrics guidelines recommend radical surgery for early-stage PVA and definitive chemoradiotherapy for advanced disease.^[10]^ Notably, therapeutic decision-making remains controversial due to the absence of randomized controlled trial evidence. Of critical concern is the statistically significant increased risk of second primary vaginal malignancies following pelvic radiotherapy, with cervical cancer survivors exhibiting higher incidence compared to nonirradiated cohorts.^[7]^

In intestinal-type vaginal adenocarcinomas, recurrent somatic alterations are observed, including TP53 inactivation, *KRAS* hotspot mutations, and constitutive activation of the mitogen-activated protein kinase (MAPK) signaling pathway.^[11]^ These genetic perturbations induce aberrant nuclear accumulation of mutant p53 protein (a surrogate biomarker quantifiable through standardized immunohistochemical assays).^[12]^ From an immunohistochemical perspective, intestinal-type adenocarcinoma exhibits distinctive characteristics, including diffuse expression of CK20 and CDX2, and frequent expression of SATB2 (a typical marker associated with colorectal origin). These tumors may also express CK7, creating a mixed immunophenotype that can complicate differentiation from metastatic colorectal adenocarcinoma. Importantly, they typically demonstrate negative reactivity for PAX8, SOX17, p16, and hormone receptors, distinguishing them from adenocarcinomas of Müllerian origin.

Clear cell adenocarcinoma represents the most distinctive subtype of vaginal adenocarcinoma, primarily due to its association with in utero DES exposure.^[10]^ Immunohistochemically, clear cell adenocarcinomas typically express CK7 and PAX8, reflecting their Müllerian origin, while being generally negative for CK20 and CDX2^[11]^. These tumors may also express hepatocyte nuclear factor-1β and napsin A, which aids in distinguishing them from other adenocarcinoma subtypes.

Endometrioid adenocarcinoma of the vagina histologically resembles endometrium, characterized by glands lined by stratified columnar epithelium with varying degrees of nuclear atypia and mitotic activity.^[13]^ Immunohistochemically, endometrioid adenocarcinomas typically express CK7, PAX8, and estrogen and progesterone receptors, while being generally negative for CK20 and CDX2^[11]^, This immunophenotype reflects their Müllerian origin and helps distinguish them from intestinal-type adenocarcinomas.

Here, we report a case of radiation-associated PVA occurring 5 years after concurrent chemoradiotherapy for stage IIA2 cervical squamous cell carcinoma. Postradiation vaginal adenocarcinoma is extremely rare. This case, without DES exposure or human papillomavirus infection, importantly demonstrated positive immunohistochemical expression of CK8/18 and CK7, while being negative for Pax-8, ER, P16, PAX8, PR, and CEA, with wild-type P53, excluding cervical adenocarcinoma and intestinal-type adenocarcinoma.

Immunohistochemistry plays a crucial role in the diagnosis and classification of vaginal adenocarcinomas, helping determine their histogenesis and distinguish them from metastatic disease. The immunohistochemical features vary by histological subtype, reflecting the diverse cellular origins of these tumors.

The rarity and histological heterogeneity of PVA present diagnostic and therapeutic challenges. Unlike common DES-related clear cell carcinomas or intestinal-type adenocarcinomas (characteristically harboring KRAS/TP53 mutations and CDX2/SATB2 positivity), this case represents radiation-associated PVA occurring 5 years after definitive chemoradiotherapy for cervical cancer. Its unique immunophenotype (CK7+, CK8/18+, PAX8−, ER−, PR−, p53 wild-type) not only excludes Müllerian origin (endometrioid/clear cell type) or metastatic colorectal adenocarcinoma but also suggests radiation-induced secondary carcinogenesis of vaginal epithelium. This finding expands our understanding of potential PVA etiology and emphasizes the importance of long-term monitoring for radiation-induced second malignancies.

Although radical surgery remains the preferred option for localized PVA, this case highlights the therapeutic dilemma posed by prior chemoradiotherapy, necessitating future integration of genomics (such as MAPK pathway analysis) for targeted therapy exploration. Moreover, precise application of immunohistochemical panels (PAX8, CDX2, p53, and hepatocyte nuclear factor-1β) remains crucial for distinguishing primary from metastatic adenocarcinomas, particularly for molecular typing of atypical cases.

## 4. Conclusion

This case provides direct evidence of radiation-associated vaginal adenocarcinoma following definitive chemoradiotherapy for cervical cancer. The tumor’s non-Müllerian, non-intestinal immunophenotype (CK7+, CK8/18+, PAX8−, ER−, and PR−), absence of conventional driver mutations (TP53/KRAS wild-type), and temporal association with prior radiation therapy (median latency: 5 years) collectively support radiation-induced microenvironmental dysregulation as a key driver of carcinogenesis. These findings reveal the dual role of radiation therapy in cancer treatment: serving both as a therapeutic cornerstone and a potential carcinogen.

For patients with previous pelvic radiation exposure, rigorous posttreatment surveillance protocols (such as annual vaginal cytology and targeted biopsies) should be established for early detection of secondary malignancies. Future research should elucidate radiation dose–response relationships, explore predictive biomarkers (such as MAPK pathway alterations), and optimize treatment strategies that balance therapeutic efficacy with long-term safety.

## Author contributions

**Conceptualization:** Guangyang Xing.

**Resources:** Xiaotong Sun.

**Supervision:** Cui Zhang, Xiaotong Sun.

**Writing – original draft:** Guangyang Xing.

**Writing – review & editing:** Guangyang Xing, Yani Lu, Cui Zhang.
